# A case of spontaneously ruptured hepatic angiosarcoma resected after transcatheter arterial embolization

**DOI:** 10.1093/jscr/rjac406

**Published:** 2022-09-27

**Authors:** Toshiaki Kashiwadate, Yasuyuki Hara, Eiji Hashizume, Akiko Nishida, Michiaki Unno, Takashi Kamei

**Affiliations:** Department of Surgery, Tohoku University Graduate School of Medicine, Sendai, Miyagi, Japan; Department of Surgery, Nihonkai General Hospital, Sakata, Yamagata, Japan; Department of Surgery, Nihonkai General Hospital, Sakata, Yamagata, Japan; Department of Pathology, Nihonkai General Hospital, Sakata, Yamagata, Japan; Department of Surgery, Tohoku University Graduate School of Medicine, Sendai, Miyagi, Japan; Department of Surgery, Tohoku University Graduate School of Medicine, Sendai, Miyagi, Japan

## Abstract

Hepatic angiosarcoma is a very rare disease, but it has a poor prognosis. Here, we report the case of a 77-year-old man who was referred to our hospital for suspicion of hepatocellular carcinoma and cancerous peritonitis. Based on the imaging findings, a diagnosis of spontaneously ruptured hepatic hemangioma was made. Six days later, the patient was transported to the hospital in a state of shock and an emergency transarterial embolization was performed. He underwent lateral hepatic segmentectomy 7 days later. Histopathologically, he was diagnosed with hepatic angiosarcoma. Fever was observed 21 days after surgery, and computed tomography was performed. Multiple liver masses, which ware increasing rapidly, were found, and hepatic angiosarcoma recurrence was confirmed. He requested home medical care and died at home 36 days after surgery. When a tumor diagnosed as a hepatic hemangioma by imaging has ruptured, the possibility of hepatic angiosarcoma should be considered.

## INTRODUCTION

Hepatic angiosarcoma is a very rare disease with a frequency of 0.04–2% among primary malignant tumors of the liver; however, it is considered to have a poor prognosis [1–3]. It is often associated with hemochromatosis and exposures to vinyl chloride monomer, thorium dioxide, arsenic compounds and radiation [3, 4]. However, in most cases unrelated to them, clinical diagnosis is usually challenging due to nonspecific clinical signs and symptoms. The most common symptoms are abdominal pain, weakness, fever and weight loss, which are also found in other types of tumors [1, 5, 6]. Hepatectomy is beneficial when the lesion is confined to a lobe of the liver without metastases, and long-term survival after resection has been occasionally reported [7]. However, most hepatic angiosarcomas are unresectable at diagnosis.

Here, we report a case of spontaneously ruptured hepatic angiosarcoma resected after transcatheter arterial embolization, which was challenging to diagnose preoperatively.

## CASE REPORT

**Figure 1:**
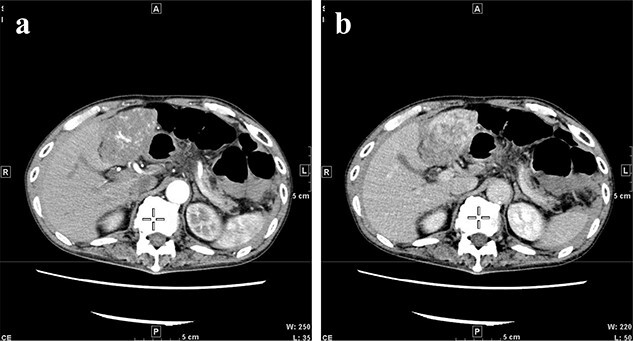
CT at initial admission points out a tumor with a maximum diameter of 8 cm in the lateral hepatic region and ascites around the liver. Dynamic CT shows blood vessel-like early staining inside the tumor, and the contrast effect gradually enhances. (**a**) Arterial phase, (**b**) equilibrium phase. CT, computed tomography.

**Figure 2:**
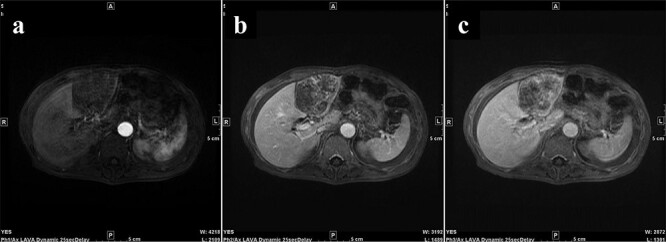
During Gd-EOB-DTPA-enhanced MRI at the time of initial hospitalization, the contrast effect of the tumor gradually increased from the peripheral part toward the hepatocyte phase from the arterial phase. (**a**) Arterial phase, (**b**) portal phase, (**c**) hepatocyte phase. Gd-EOB-DTPA, gadolinium ethoxybenzyl diethylenetriamine pentaacetic acid; MRI, magnetic resonance imaging.

**Figure 3:**
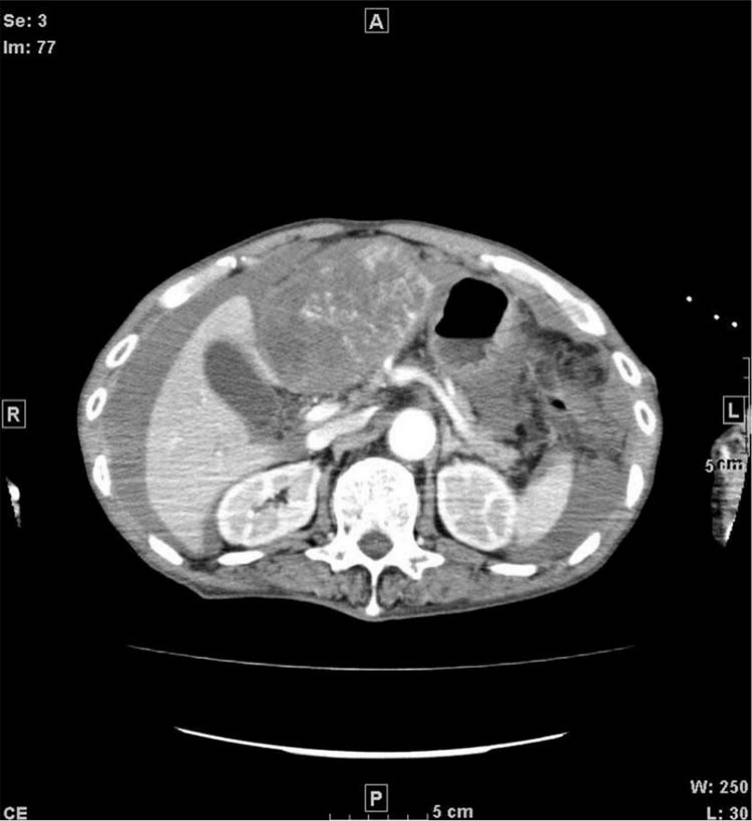
CT when the patient was transported in a state of shock shows increased bloody ascites but no apparent extravasation. CT, computed tomography.

**Figure 4:**
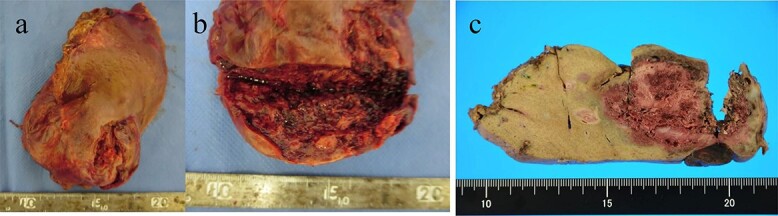
(**a**, **b**) A macroscopic image of the post-ruptured tumor (7 × 4 cm) in the left lateral segment. (**c**) After formalin fixation. The cut surface is white, the edges are brown and the boundaries are unclear.

**Figure 5:**
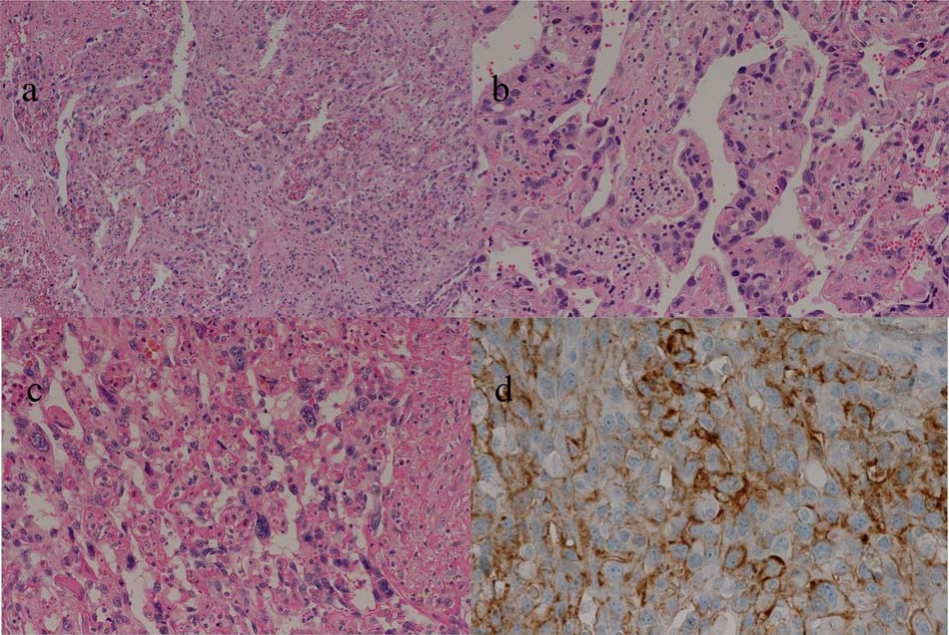
(**a–c**) Sections of the white and brown parts of the margin reveal viable tumors, highly atypical spindle-shaped cells, arranged like sinusoidal vessels and partly solid, with many mitotic figures. There is no capsule, and the tumor is shown infiltrating the surrounding liver parenchyma. (**d**) Immunostaining shows CD34 (partially positive), and the patient is diagnosed as having hepatic angiosarcoma.

**Figure 6:**
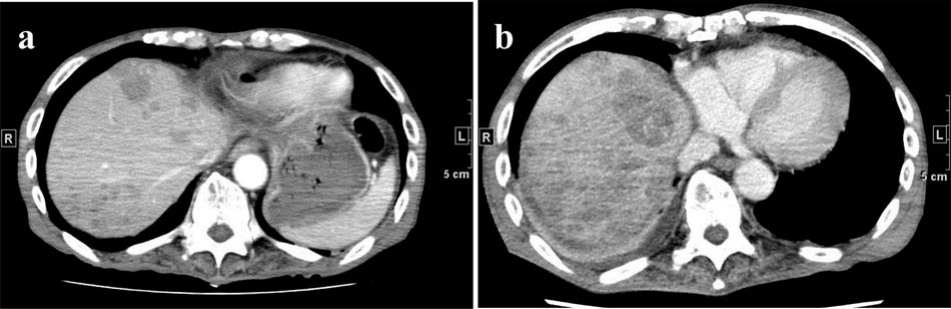
(**a**) CT on Day 21 after surgery. Multiple tumors are found in the liver. (**b**) CT re-examination performed 27 days after the operation shows multiple rapidly increasing liver masses. CT, computed tomography.

A 77-year-old man was referred to the Department of Gastroenterology at our hospital for suspicion of hepatocellular carcinoma and cancerous peritonitis. He was scheduled to be hospitalized for a thorough examination at a later date. However, 3 days after his first visit, he returned to the Emergency Department with a complaint of abdominal distension. He was hospitalized on the same day with a diagnosis of intra-abdominal bleeding due to a ruptured tumor in the lateral section of the liver. Computed tomography (CT) and magnetic resonance imaging (MRI) revealed a tumor with a maximum diameter of 8 cm in the lateral hepatic region and ascites around the liver and in the pelvic floor. Dynamic CT showed blood vessel-like early staining inside the tumor, and the contrast effect gradually enhanced (Fig. 1). During gadolinium ethoxybenzyl diethylenetriamine pentaacetic acid-enhanced MRI, this contrast effect gradually increased from the peripheral part toward the hepatocyte phase from the arterial phase (Fig. 2). Levels of tumor markers (alpha-fetoprotein, protein induced by vitamin K absence or antagonist-II, carcinoembryonic antigen and carbohydrate antigen 19–9) were within the normal range (Table 1). Based on the imaging findings, a diagnosis of spontaneously ruptured hepatic hemangioma was made. Since the patient’s general condition was stable and there were no findings suggestive of active bleeding, he decided to undergo elective surgery. Thus, he was not requested to continue being hospitalized and was discharged. Six days after discharge, he was transported to the emergency room in a state of shock and was hospitalized the same day. With the diagnosis of hemorrhagic shock due to re-rupture of the liver tumor (Fig. 3), emergency transarterial embolization (TAE) was performed. No apparent extravasation was observed on angiography, and A3 was embolized. Seven days later, he underwent left lateral hepatic segmentectomy. The postoperative course was generally good. The post-ruptured tumor was found in the left lateral segment, the cut surface was white, the edges were brown and the boundaries were unclear (Fig. 4). Histopathological examination revealed that sections of the white and brown parts of the margin were viable tumors, showing highly atypical spindle-shaped cells, arranged like sinusoidal vessels and partly solid, and there were many mitotic figures. There was no capsule, and the tumor infiltrated the surrounding liver parenchyma (Fig. 5a–c). Immunostaining showed CD31 (+), CD34 (partially positive), CK-CAM5.2 (−), α-SMA (−), HMB45 (indeterminate) and p53 (+), and the patient was diagnosed with hepatic angiosarcoma (Fig. 5d). Fever was observed 21 days after surgery, and CT was performed, revealing multiple masses in the liver (Fig. 6a). Another CT scan was performed 27 days postoperatively, which suggested that the liver masses were rapidly increasing; hepatic angiosarcoma recurrence was then confirmed (Fig. 6b). The patient requested the best supportive care at home and was discharged 31 days after the operation. He died at home 36 days after surgery.

**Table 1:** Laboratory data on admission.

RPR	(−)	T-bil	1.0 mg/dl	Na	141 mEq/l
TP	(−)	D-bil	0.2 mg/dl	K	4.2 mEq/l
HBs Ag	0.001 IU/ml	ALP	246 U/l	Cl	104 mEq/l
HCV Ab	0.1	γ-GTP	24 U/l	TG	68 mg/dl
HIV Ab	(−)	AST	38 U/l	T-CHO	146 mg/dl
WBC	6030/μl	ALT	20 U/l	Glu	111 mg/dl
RBC	228 × 10^4^/μl	LDH	215 U/l	HbA1c	5.4%
Hb	7.7 g/dl	ChE	156 U/l	ICGR15	5.2%
PLT	18.6 × 10^4^/μl	BUN	19.3 mg/dl		
PT	85.9%	Cre	0.69 mg/dl	AFP	1.6 ng/ml
PT-INR	1.08	TP	6.8 g/dl	PIVKA-II	16mAU/ml
		Alb	4.0 g/dl	CEA	2.0 ng/ml
		CRP	1.85 mg/dl	CA19–9	5.14 U/ml

## DISCUSSION

The risk of rapid progression and metastasis is high among patients with hepatic angiosarcoma, and the mean survival time is reported to be 5.5–6.0 months [5, 8]. Our patient had a rapid course and died in just 36 days after surgery. The etiology of 75% of primary hepatic angiosarcomas is unknown, and although the remaining 25% are believed to be related to hemochromatosis and exposure to vinyl chloride monomer, thorium dioxide, arsenic compounds and radiation [3, 4], in our case there was no history of exposure to these substances. Because exposure to these agents has become uncommon in modern practice [9], it is not surprising that none of these environmental risk factors existed in this case.

Hepatectomy is the treatment of choice for resectable hepatic angiosarcoma, but there are many cases with distant metastasis at the time of diagnosis, and radical resection is possible in few cases [8]. Although paclitaxel is effective in treating unresectable angiosarcoma [10], there is no established chemotherapy. Our patient was transported to the hospital in a state of shock due to tumor rupture, and although his general condition was stabilized by TAE, it was difficult to perform chemotherapy or follow-up observation in this condition. Therefore, it was challenging to consider another option other than surgery.

Hepatic angiosarcoma is sometimes difficult to distinguish from hepatic hemangioma on imaging [11, 12]. The probability of a spontaneous tumor rupture in the natural course is reported to be 15–27% for hepatic angiosarcoma compared with <1% for hepatic hemangioma [2, 13–15]. At the time of the outpatient visit, our patient was diagnosed with spontaneously ruptured hepatic hemangioma based on the imaging findings, and elective surgery was planned. However, given the differences in the frequency of spontaneous rupture, hepatic angiosarcoma should have been more strongly suspected.

In conclusion, although spontaneous rupture of a tumor diagnosed as hepatic hemangioma by imaging is very rare, the possibility of hepatic angiosarcoma should be considered.
